# In situ cryo-electron microscopy and tomography of cellular and organismal samples

**DOI:** 10.1016/j.sbi.2025.103076

**Published:** 2025-06-04

**Authors:** Parijat Majumder, Peijun Zhang

**Affiliations:** 1 Diamond Light Source, Harwell Science and Innovation Campus, Didcot, OX11 0DE, UK; 2 Division of Structural Biology, Nuffield Department of Medicine, University of Oxford, Oxford, OX3 7BN, UK

## Abstract

As cryo-electron microscopy (cryo-EM) and cryo-electron tomography (cryo-ET) continue to advance, the ability to visualize cellular and organismal structures with unprecedented clarity is redefining the landscape of structural biology. Breakthroughs in imaging technology, sample preparation and image processing now enable the detailed elucidation of cellular architecture, macromolecular organization, and dynamic biological processes at sub-nanometer resolution. Recent methodological advances have propelled the field to new frontiers, facilitating the investigation of complex biological questions across scales—from macromolecular complexes to organism-wide structural insights. This review explores rapidly emerging trends, highlights key innovations that are pushing the boundaries of in situ structural biology, and addresses persistent challenges in expanding the applicability of cryo-EM and cryo-ET across diverse biological systems.

## Introduction

Cryo-EM and cryo-ET have emerged as indispensable tools in structural biology, that enable the determination of three-dimensional (3D) structures of biological systems in near-native states. The “resolution revolution” in cryo-EM has enabled visualization of isolated biomolecules in unprecedented detail. Simultaneously, cryo-ET has earned powerful capabilities for elucidating the internal architecture of cells and tissues [1,2].

Over the past decade, these techniques have been significantly enhanced by the integration of complementary approaches [2]. Focused ion beam (FIB) milling has facilitated the preparation of thin lamellae from thick biological samples, enabling high-resolution imaging [3]. Correlative light and electron microscopy (CLEM) has bridged functional and structural imaging, combining fluorescence-based localization with cryo-EM and cryo-ET [4,5]. Furthermore, advancements in deep learning have powered data processing and structural reconstruction [6]. These innovations have significantly expanded the scope of cryo-EM and cryo-ET in cellular and organismal research, supporting applications across diverse fields, spanning organelle dynamics, structural virology, host–pathogen interactions, neurodegeneration, and phase separation.

**Figure 1:**
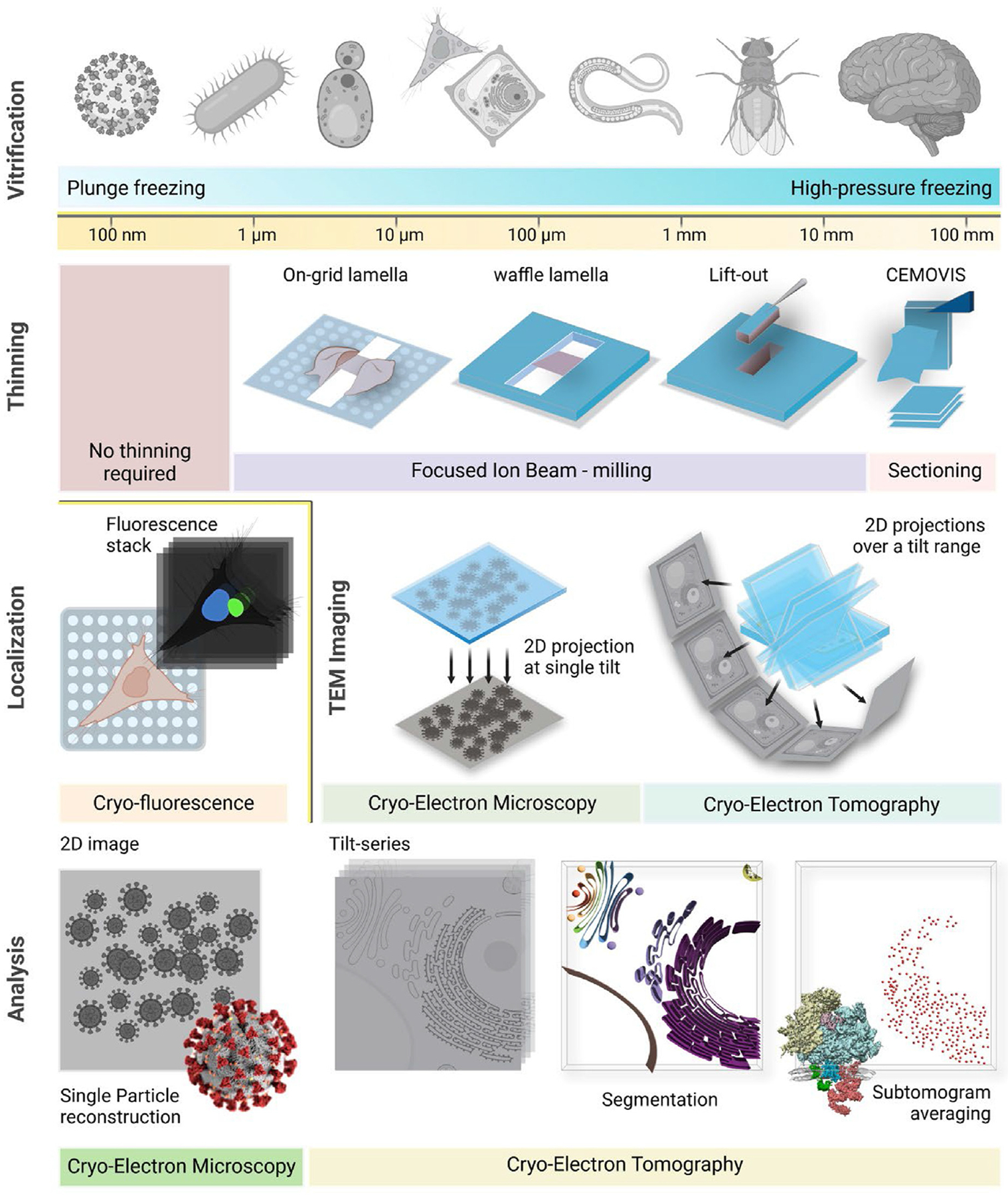
Overview of the cryo-EM and cryo-ET methods for cellular and organismal samples (Vitrification) Cellular and organismal samples vary in size, ranging from less than a hundred nanometres to several hundred millimetres. Representative specimens, from left to right, include viruses, bacteria, yeast, animal and plant cells, worms, fruit flies, and the human brain. The selection of vitrification methods is dictated by specimen size. Plunge freezing is suitable for samples with a thickness of less than ~10 μm, whereas high-pressure freezing is more effective for thicker specimens, currently up to a few hundred micrometres (Thinning) This step is necessary for samples that are thicker than ~ 1 μm. Focused ion beam (FIB) milling can be used for on-grid lamella preparation, waffle lamellae and for lift-out, with the choice of method dictated by sample geometry. Cryo-sectioning (CEMOVIS) offers an alternative thinning route for high pressure frozen samples (Localization) Cryo-fluorescence imaging of vitrified specimens enables the identification and localization of regions of interest, guiding targeted thinning or transmission electron microscopy (TEM) imaging (TEM imaging) Cryo-EM involves acquisition of 2D projection images at a single angle, while cryo-ET involves acquisition of 2D images over a range of sample tilt angles. (Analysis) In cryo-EM, 2D projections are conventionally processed using the single-particle analysis pipeline to achieve high-resolution 3D reconstructions. In cryo-ET, tilt projection series are reconstructed into 3D volumes or tomograms, enabling ultrastructural elucidation through segmentation or the generation of 3D macromolecular reconstructions via subtomogram averaging. (This figure is partially prepared in BioRender).

**Figure 2:**
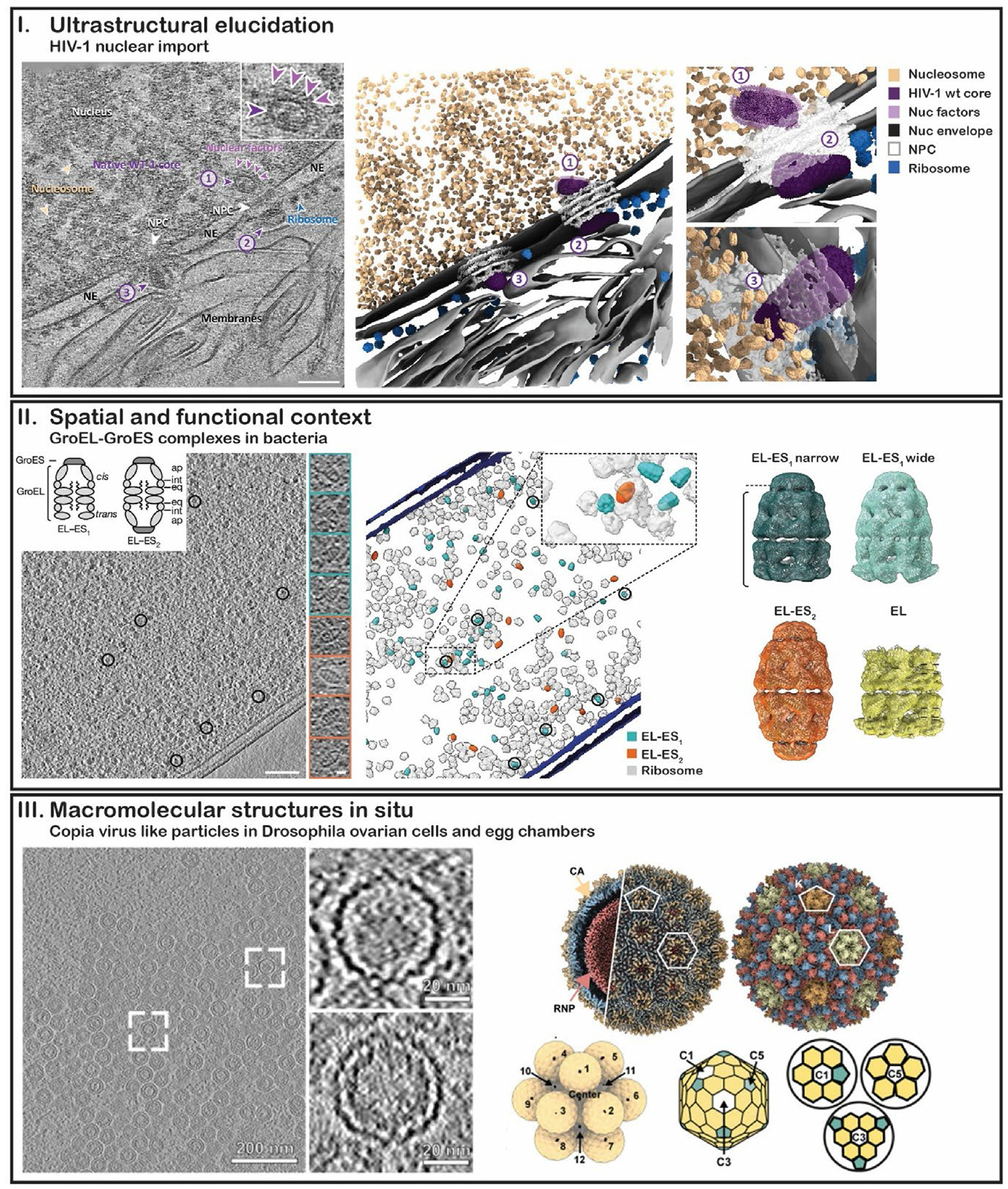
Latest examples of in situ cryo-ET applications I. Ultrastructural Elucidation. A representative tomographic slice (left panel) and the corresponding segmented volume (middle and right panels) illustrate the nuclear import of wild-type HIV-1 cores [41]. Three cores are identified and marked with purple arrowheads and numbers: No.1, an imported tube-shaped core with discernible surrounding densities (enlarged in the inset); No.2, a docked cone-shaped core with its wide end positioned on the nuclear pore complex (NPC); and No.3, a cone-shaped core traversing the NPC, with its narrow end inside the nucleus. The NPC, ribosomes, nucleosomes, and prominent nuclear factors are labelled. The nucleus, nuclear envelope (NE), and membranes are annotated accordingly. Scale bar: 100 nm. II. Spatial and Functional Context. A representative tomographic slice of a heat shock-exposed *E. coli* cell (left panel) and the corresponding three-dimensional rendering of macromolecular complexes (right panel) illustrate the spatial organization of GroEL–GroES complexes and ribosomes in situ [64]. GroEL–GroES complexes are indicated by black open circles. Scale bar: 100 nm. A schematic representation of asymmetrical (EL-ES_1_) and symmetrical (EL-ES_2_) GroEL–GroES complexes is shown in the top-left inset. To the right of the tomographic slice, a gallery presents central subtomogram slices of EL-ES_1_ and EL-ES_2_ complexes in a side view. Scale bar: 10 nm. The right panel shows subtomogram averages of GroEL–GroES complexes in different conformations and stoichiometries. III. Macromolecular Structures *In Situ*. A representative tomographic slice (left panel) of a nuclear *copia* cluster in *Drosophila* ovarian cells shows virus-like particles (VLPs) [78]. Scale bar, 200 nm. Rectangles highlight a putative immature VLP and a putative mature VLP, for which the corresponding zoomed-in views are shown in the right inset. Scale bar, 20 nm. The right panel (top) displays a 7.7 Å resolution subtomogram average of the entire *copia* CA, alongside an AlphaFold-Multimer and MDFF-based model of the complete CA structure. The right panel (bottom) presents a rendering of a repeat unit from a nuclear *copia* cluster, along with schematic representations of different environments within an icosahedral lattice.

This review provides an overview of the methods (Figure 1) and latest applications (Figure 2) of cryo-EM and cryo-ET in cellular and organismal research. By examining recent developments and addressing current challenges, it highlights the transformative potential of these techniques for in situ structural studies.

## Methods for Cryo-EM and Cryo-ET of cellular and organismal samples

### Sample preparation techniques

For high-resolution imaging, samples must be vitrified to preserve their biological structures in a near-native state [7]. Vitrification involves rapid freezing, typically achieved through immersion in cryogens such as liquid ethane or ethane-propane mixtures. This process prevents ice crystal formation, resulting in amorphous, glass-like ice that retains the structural properties of liquid water [7].

Cellular samples can be broadly categorized as adherent (e.g., mammalian cells) or suspended (e.g., bacteria, yeast, algae, or certain mammalian cells). Adherent specimens are typically cultured directly on cryo-EM grids, while suspended specimens are applied to grids similarly to isolated biomolecules. The choice of vitrification method depends on sample type and thickness. Plunge freezing is suitable for specimens thinner than ~10 μm, although cryoprotectants are often required for samples exceeding ~5 μm [8]. For thicker specimens, such as organismal samples or tissues, high-pressure freezing is necessary to ensure effective vitrification and structural preservation [9]. These specimens can be vitrified in various carriers and are processed accordingly [8].

For imaging via modern transmission electron microscopy (TEM), samples must be thinner than 500 nm to achieve electron transparency. As many cellular and organismal samples exceed this limit in dense regions, such as near nuclei, imaging is often limited to thinner peripheral areas or requires methods to create electron-transparent windows.

### Sample thinning

Cells and tissues can sometimes be rendered electron-transparent using mild purification methods—such as unroofing or cell permeabilization—although these approaches risk compromising sample integrity. To thin samples without chemical perturbation, cryogenic sectioning was initially developed, enabling cryo-EM of vitreous sections (CEMOVIS) [10,11]. In this technique, an ultramicrotome equipped with a cold diamond knife is used to slice ultrathin sections, which are then transferred onto cryo-EM grids for imaging. Although CEMOVIS has facilitated the visualization of large specimens, including tissues and multicellular organisms, artifacts induced by section compression frequently limit its utility.

In recent years, cryo-FIB milling has gained attraction for sample thinning [3]. This method employs ion beam–directed ablation to generate lamellae thinner than 300 nm and can be tailored for both plunge-frozen and high-pressure frozen sample types [12]. Despite its considerable utility, cryo-FIB milling is currently hindered by efficiency constraints due to time-consuming procedures. However, the adoption of plasma ion sources and automated pipelines are expected to significantly enhance its throughput [13]. Moreover, hybrid approaches that combine sectioning and cryo-FIB milling are now emerging [14]. By leveraging the strengths of both methods, these integrated strategies promise to expand the versatility of sample preparation, thereby enabling high-resolution imaging across a broader spectrum of biological specimens.

### Feature localization

As the field advances to address increasingly complex questions in cellular and organismal biology, there is an increasing demand for spatiotemporal precision in cryo-EM and cryo-ET. Immunolabeling and the application of electron dense probes have long served as strategies to enable the in situ localization of cellular events and biomolecules [15,16]. However, the integration of fluorescent markers with cryo-imaging has led to correlative light and cryo-electron microscopy (cryo-CLEM) as a powerful means to enhance spatial and molecular precision [4,17].

In a typical cryo-CLEM workflow, vitrified samples are first imaged using cryogenic light (fluorescence) microscopy (cryo-LM) to identify features of interest. These fluorescence images are then aligned with scanning or transmission electron microscopy images via coordinate transformation, allowing seamless cross–modality correlation [18]. For events located in the thin sample areas, fluorescence guides area selection for high-resolution imaging. For events in thick regions, it guides cryo-sectioning or lamella preparation via FIB milling, and subsequent targeted imaging.

Although cryo-CLEM significantly enhances feature localization, the resolution attainable with cryo-LM remains lower than that of conventional, non-cryogenic systems. The adaptation of super-resolution fluorescence techniques—such as stimulated emission depletion (STED), structured illumination microscopy (SIM), photoactivatable localization microscopy (PALM), and stochastic optical reconstruction microscopy (STORM)—to cryogenic conditions has introduced single-molecule sensitivity and specificity [19]. However, workflows involving multiple instrument transfers increase the risk of sample contamination and devitrification due to exposure outside of vacuum. As a practical solution, integrated cryogenic instruments have been developed that incorporate fluorescence imaging directly within FIB chambers [20]. While the resolution of commercially available integrated setups is currently lower than that of stand-alone cryo-LM systems, they enable direct signal verification on lamellae, thereby improving the reliability of downstream analyses.

### Imaging cellular and organismal samples

Cryo-EM and cryo-ET are both powerful techniques for structural studies of cellular and organismal samples, with the choice of method depending on the research objectives. These methods differ fundamentally in data acquisition strategies and the type of information they provide.

Cryo-EM captures single projection images at fixed sample stage angles, producing two-dimensional (2D) representations of the specimen. This approach underlies single-particle analysis (SPA), where individual projections provide randomly oriented views of purified biomolecules. Through computational alignment and averaging of these images, high-resolution three-dimensional (3D) structures can be reconstructed [21].

In contrast, cryo-ET acquires a series of projection images across a defined tilt range of the specimen, which are then computationally reconstructed into 3D volumes or tomograms [22,23]. To analyse recurring structural patterns within these tomograms, coordinates are selected using either template-based or template-free approaches [24,25]. This is followed by subvolume or subtomogram averaging (STA), which aligns and averages the extracted subvolumes to generate 3D reconstructions [26,27]. Consequently, cryo-ET enables the in situ visualization of macromolecular assemblies and cellular ultrastructure, preserving their native spatial and contextual organization.

While technological advancements have markedly improved the quality of cryo-EM data—enabling the visualization of cellular features previously beyond detection—the resolution achievable through STA continues to lag behind that attained via SPA. This limitation is largely due to the intrinsically low signal-to-noise ratio of cryo-ET data, which results from the densely packed cellular environment and the necessity of distributing the electron dose over a wide tilt range.

To mitigate these constraints, a range of hybrid data acquisition and processing strategies has emerged to maximize structural information [28–30]. In the cellular context, a notable development is hybrid single-particle tomography, in which a high-dose, untilted image is first acquired, followed by a tilt series of the same field of view [28,29]. This facilitates the determination of 3D structures, while preserving information on particle orientations and positions. Other approaches, such as 2D template matching, involve comparing projections of known structures against untilted images of thin cellular specimens, enabling the identification of macromolecular complexes without requiring tilt-series acquisition [31,32]. By combining the high-resolution potential of SPA with the spatial contextualization afforded by cryo-ET, these hybrid cryo-EM approaches offer a powerful framework for in situ structural biology—providing the possibility of achieving resolutions that surpass those attainable by conventional STA alone.

Parallel advances in image processing—particularly the integration of deep learning for structural pattern recognition, denoising, classification, and segmentation—have significantly enhanced the analytical capabilities of cryo-ET. A detailed discussion of these developments lies beyond the scope of the present review; readers are instead referred to the following comprehensive review for further information [6].

## Applications of Cryo-EM and Cryo-ET

### Subcellular architecture

Cryo-imaging techniques are essential for elucidating subcellular architecture and characterizing ultrastructural features across diverse biological contexts. Given the complexity of cellular interiors, research has traditionally focused on readily identifiable structures such as membranes, cytoskeletal elements, vesicles, and internalized or isolated pathogens. By segmenting these features, researchers have quantified key biophysical parameters, including membrane curvature, inter-feature distances, and filament persistence lengths [33–38]. These measurements have substantially refined our understanding of organelle ultrastructure, inter-organelle interactions, membrane dynamics, and overall subcellular morphology.

In recent years, cryo-ET has provided unprecedented ultrastructural insights into viral infections, illustrating key processes such as viral entry [39,40], nuclear import [41,42], the formation of replication organelles [43], and the hijacking of autophagy [44]. It has also elucidated infection strategies that pathogens adopt to subvert host cellular machinery [45]. Beyond viral pathogenesis, these investigations have been instrumental in uncovering biomolecular condensates across diverse cellular contexts [46,47], underscoring their fundamental role in cellular organization and function. Additionally, they have provided critical insights into inflammasome signaling by capturing NLRP3-activated ASC complexes [48], their associated mitochondrial dynamics, and inflammation-associated condensate formation at the microtubule-organizing centre [49].

Further, ultrastructural elucidations have advanced our understanding of protein quality control [50] and autophagy, shedding light on how cells orchestrate the degradation and recycling of damaged proteins, organelles, and pathogens [51–53]. These findings have significant implications for cellular stress responses and neurodegenerative conditions.

While cryo-ET investigations of neurons have been reported previously, recent studies represent extensions of this earlier work, continuing to characterize synaptic components such as vesicles and scaffold proteins—key elements for neurotransmission and synaptic plasticity [54,55]. In contrast, major recent advancements have been made at the tissue level, where cryo-ET has provided detailed insights into native hippocampal glutamatergic synapses in transgenic mouse brains [56], as well as β-amyloid and tau pathology in postmortem Alzheimer’s disease brains [57].

Across biological kingdoms, cryo-imaging has revealed fundamental structural adaptations that drive cellular function and evolution. For instance, it has elucidated the complex cellular architecture of the Asgard archaeon [58], demonstrated how ferrosome organelles mitigate nutritional immunity [59], and uncovered a protein-shell-mediated mechanism for CO_2_ fixation in diatom pyrenoids [60]. Collectively, these findings provide a structural framework for understanding cellular adaptations from an evolutionary perspective.

### Macromolecules in cellular contexts

Imaging of cellular and organismal samples enables the structural characterization of macromolecular assemblies within their native contexts. While these methods are most effective when applied to large, abundant, high-contrast, or symmetric structures, they have significantly advanced our understanding of cellular machinery by providing unique insights into spatial organization, dynamics, and molecular mechanisms.

Historically, ribosomes have been among the most visually recognizable targets for in situ structure determination, offering invaluable insights into translation. These studies have enabled the visualization of translation dynamics under various conditions, including the effects of cancer drugs [61], ribosome collision stress [62], and chloramphenicol exposure [63].

Recent in situ analyses have expanded to other essential complexes, such as the chaperonins GroEL–GroES in bacteria [64] and TRiC (CCT) in human cells [65]. By defining their spatial organization and conformational states, these studies have provided valuable insights into protein folding mechanisms. Structural features of large assemblies, such as the nuclear pore complex basket [66] and cardiac myosin filaments [67], have also been elucidated. Additionally, cryo-ET has facilitated the visualization of centrosomal organization in *Caenorhabditis elegans* embryos [68] and identified tubulin chaperones as a distinct subset of microtubule luminal particles, suggesting a role in neuronal microtubule maintenance [69].

Viral pathogenesis has been a major area of advancement. In situ structural studies have provided mechanistic insights into SARS-CoV-2 infection [70], vaccinia virus maturation [71], rotavirus assembly [72], herpesvirus nuclear egress [73], and RNA genome packaging in bluetongue virus [74]. Native structures of the ChAdOx1 nCoV-19 vaccine product have also been resolved on the surface of transfected cells [75]. Further breakthroughs include the visualization of Ebola virus nucleocapsid assembly [76], viral factory maturation and dispersion [77], and copia virus-like particles in *Drosophila* ovarian cells and egg chambers [78].

These imaging approaches have also enhanced our understanding of host–pathogen interactions and immune defence. For example, they have clarified bacterial predation by ixotrophy [79], revealed the detailed architecture of actin networks in apicomplexans and polar tube dynamics in microsporidia [80], and unveiled the molecular organization of the human GBP1 defence complex [81].

Beyond pathogenesis, structural studies have illuminated carbon fixation strategies. The spatial arrangement of Rubisco within α- and β-carboxysomes [82–84], as well as within the pyrenoid of *Chlamydomonas reinhardtii* [85], has shed light on CO_2_-concentrating mechanisms in cyanobacteria and eukaryotic chloroplasts.

Furthermore, advances in imaging technologies and data quality have led to significant breakthroughs in resolving membrane protein complexes, such as SARS-CoV-2 vaccine spikes [75], the synaptic V-ATPase–synaptophysin complex [86], mitochondrial respiratory chain assemblies [87,88], bacterial chemoreceptor signalling arrays [89], the particulate methane monooxygenase [90], and the human prohibitin complex [91]. Notably, it is now also possible to resolve macromolecular assemblies within the relatively low-contrast environment of the nucleus. For example, this has enabled the visualization of chromatin fibers and nucleosome structures, opening new avenues for investigating three-dimensional genome organization and its regulation [92].

## Concluding remarks

Despite significant advances in cryo-imaging over the past decade, its routine application remains hindered by several challenges. Sample preparation, target identification, and data interpretation continue to pose major obstacles. Although cryo-FIB milling has facilitated sample thinning, further refinement of vitrification and milling protocols is needed to accommodate the heterogeneity, morphology, and complexity of biological specimens, as well as to enhance throughput. Additionally, many intracellular structures remain uncharacterized, with densely packed cytoplasm and featureless nuclei contributing to poor signal-to-noise ratios and complicating structural interpretation. While cryo-CLEM supports targeting, its effectiveness is limited by low resolution, reliance on fluorescent labelling, and labour–intensive correlation workflows.

Looking forward, hybrid strategies such as integrating cryo-EM/ETwith volume EM offer exciting possibilities for bridging molecular detail with broader cellular context [93]. Artificial intelligence, though currently applied in limited ways—primarily in denoising and segmentation—holds substantial promise for the field [94,95]. As AI technologies advance, especially in areas like real-time targeting, automated acquisition, and biologically meaningful interpretation, they may drive paradigm-shifting changes in cryo-EM/ET. Tools such as SPACE tomo [96] and the emergence of annotated tomogram databases [97] mark important steps toward high-resolution cellular mapping and visual proteomics.

## Data Availability

No data was used for the research described in the article.

## References

[1] Young LN, Villa E: Bringing structure to cell biology with cryo-electron tomography. Annu Rev Biophys 2023, 52:573–595. 37159298 10.1146/annurev-biophys-111622-091327 PMC10763975

[2] Nogales E, Mahamid J: Bridging structural and cell biology with cryo-electron microscopy. Nature 2024, 628:47–56. 38570716 10.1038/s41586-024-07198-2 PMC11211576

[3] Marko M, Hsieh C, Schalek R, Frank J, Mannella C: Focusedion-beam thinning of frozen-hydrated biological specimens for cryo-electron microscopy. Nat Methods 2007, 4: 215–217. 17277781 10.1038/nmeth1014

[4] Sartori A, Gatz R, Beck F, Rigort A, Baumeister W, Plitzko JM: Correlative microscopy: bridging the gap between fluorescence light microscopy and cryo-electron tomography. J Struct Biol 2007, 160:135–145. 17884579 10.1016/j.jsb.2007.07.011

[5] Pierson JA, Yang JE, Wright ER: Recent advances in correlative cryo-light and electron microscopy. Curr Opin Struct Biol 2024, 89, 102934. 39366119 10.1016/j.sbi.2024.102934 PMC11602379

[6] Watson AJI, Bartesaghi A: Advances in cryo-ET data processing: meeting the demands of visual proteomics. Curr Opin Struct Biol 2024, 87, 102861. 38889501 10.1016/j.sbi.2024.102861 PMC11283971

[7] Dubochet J, Adrian M, Chang JJ, Homo JC, Lepault J, McDowall AW, Schultz P: Cryo-electron microscopy of vitrified specimens. Q Rev Biophys 1988, 21:129–228. 3043536 10.1017/s0033583500004297

[8] McDonald K: Cryopreparation methods for electron microscopy of selected model systems. Methods Cell Biol 2007, 79: 23–56. 17327151 10.1016/S0091-679X(06)79002-1

[9] Dahl R, Staehelin LA: High-pressure freezing for the preservation of biological structure: theory and practice. J Electron Microsc Tech 1989, 13:165–174. 2685196 10.1002/jemt.1060130305

[10] Al-Amoudi A, Norlen LP, Dubochet J: Cryo-electron microscopy of vitreous sections of native biological cells and tissues. J Struct Biol 2004, 148:131–135. 15363793 10.1016/j.jsb.2004.03.010

[11] Chlanda P, Sachse M: Cryo-electron microscopy of vitreous sections. Methods Mol Biol 2014, 1117:193–214. 24357365 10.1007/978-1-62703-776-1_10

[12] Schiotz OH, Klumpe S, Plitzko JM, Kaiser CJO: Cryo-electron tomography: en route to the molecular anatomy of organisms and tissues. Biochem Soc Trans 2024, 52:2415–2425. 39641594 10.1042/BST20240173 PMC11668301

[13] Berger C, Dumoux M, Glen T, Yee NB, Mitchels JM, Patakova Z, Darrow MC, Naismith JH, Grange M: Plasma FIB milling for the determination of structures in situ. Nat Commun 2023, 14: 629. 36746945 10.1038/s41467-023-36372-9 PMC9902539

[14] Glynn C, Smith JLR, Case M, Csöndör R, Katsini A, Sanita ME, Glen TS, Pennington A, Grange M: Charting the molecular landscape of neuronal organisation within the hippocampus using cryo electron tomography. bioRxiv 2024, 10.1101/2024.10.14.617844:2024.2010.2014.617844.

[15] Clayworth K, Gilbert M, Auld V: Whole-larva cryosectioning and immunolabeling of Drosophila larvae. Cold Spring Harb Protoc 2024, 2024, 10.1101/pdb.prot108161. 37399178

[16] Wang Q, Mercogliano CP, Lowe J: A ferritin-based label for cellular electron cryotomography. Structure 2011, 19: 147–154. 21300284 10.1016/j.str.2010.12.002

[17] Schwartz CL, Sarbash VI, Ataullakhanov FI, McIntosh JR, Nicastro D: Cryo-fluorescence microscopy facilitates correlations between light and cryo-electron microscopy and reduces the rate of photobleaching. J Microsc 2007, 227:98–109. 17845705 10.1111/j.1365-2818.2007.01794.x

[18] Arnold J, Mahamid J, Lucic V, de Marco A, Fernandez JJ, Laugks T, Mayer T, Hyman AA, Baumeister W, Plitzko JM: Site-specific cryo-focused ion beam sample preparation guided by 3D correlative microscopy. Biophys J 2016, 110:860–869. 26769364 10.1016/j.bpj.2015.10.053 PMC4775854

[19] Dahlberg PD, Moerner WE: Cryogenic super-resolution fluorescence and electron microscopy correlated at the nanoscale. Annu Rev Phys Chem 2021, 72:253–278. 33441030 10.1146/annurev-physchem-090319-051546 PMC8877847

[20] Li W, Lu J, Xiao K, Zhou M, Li Y, Zhang X, Li Z, Gu L, Xu X, Guo Q, : Integrated multimodality microscope for accurate and efficient target-guided cryo-lamellae preparation. Nat Methods 2023, 20:268–275. 36646896 10.1038/s41592-022-01749-z PMC9911353

[21] Singer A, Sigworth FJ: Computational methods for single-particle electron cryomicroscopy. Annu Rev Biomed Data Sci 2020, 3:163–190. 34485850 10.1146/annurev-biodatasci-021020-093826 PMC8412055

[22] Dierksen K, Typke D, Hegerl R, Baumeister W: Towards automatic electron tomography II. Implementation of autofocus and low-dose procedures. Ultramicroscopy 1993, 49: 109–120.

[23] Wan W, Briggs JA: Cryo-electron tomography and subtomogram averaging. Methods Enzymol 2016, 579: 329–367. 27572733 10.1016/bs.mie.2016.04.014

[24] Bohm J, Frangakis AS, Hegerl R, Nickell S, Typke D, Baumeister W: Toward detecting and identifying macromolecules in a cellular context: template matching applied to electron tomograms. Proc Natl Acad Sci U S A 2000, 97: 14245–14250. 11087814 10.1073/pnas.230282097 PMC18903

[25] Wu X, Zeng X, Zhu Z, Gao X, Xu M: Template-based and template-free approaches in cellular cryo-electron tomography structural pattern mining. In Computational biology. Edited by Husi H; 2019, 10.15586/computationalbiology.2019.ch11. 31815400

[26] Forster F, Hegerl R: Structure determination in situ by averaging of tomograms. Methods Cell Biol 2007, 79:741–767. 17327182 10.1016/S0091-679X(06)79029-X

[27] Zhao C, Lu D, Zhao Q, Ren C, Zhang H, Zhai J, Gou J, Zhu S, Zhang Y, Gong X: Computational methods for in situ structural studies with cryogenic electron tomography. Front Cell Infect Microbiol 2023, 13, 1135013. 37868346 10.3389/fcimb.2023.1135013 PMC10586593

[28] Song K, Shang Z, Fu X, Lou X, Grigorieff N, Nicastro D: In situ structure determination at nanometer resolution using TYGRESS. Nat Methods 2020, 17:201–208. 31768058 10.1038/s41592-019-0651-0 PMC7004880

[29] Sanchez RM, Zhang Y, Chen W, Dietrich L, Kudryashev M: Subnanometer-resolution structure determination in situ by hybrid subtomogram averaging - single particle cryo-EM. Nat Commun 2020, 11:3709. 32709843 10.1038/s41467-020-17466-0 PMC7381653

[30] Calcraft T, Rosenthal PB: Cryogenic electron microscopy approaches that combine images and tilt series. Microscopy (Oxf) 2022, 71:i15–i22. 35275182 10.1093/jmicro/dfab053 PMC8855521

[31] Rickgauer JP, Grigorieff N, Denk W: Single-protein detection in crowded molecular environments in cryo-EM images. eLife 2017, 6. 10.7554/eLife.25648 PMC5453696 28467302

[32] Lucas BA, Himes BA, Xue L, Grant T, Mahamid J, Grigorieff N: Locating macromolecular assemblies in cells by 2D template matching with cisTEM. eLife 2021, 10. 10.7554/eLife.68946 PMC8219381 34114559

[33] Trachtenberg S, Hammel I: Determining the persistence length of biopolymers and rod-like macromolecular assemblies from electron microscope images and deriving some of their mechanical properties. Microscopy: Sci Technol Appli Educ 2010, 3:1690–1695.

[34] Martinez-Sanchez A, Garcia I, Asano S, Lucic V, Fernandez JJ: Robust membrane detection based on tensor voting for electron tomography. J Struct Biol 2014, 186:49–61. 24625523 10.1016/j.jsb.2014.02.015

[35] Salfer M, Collado JF, Baumeister W, Fernandez-Busnadiego R, Martinez-Sanchez A: Reliable estimation of membrane curvature for cryo-electron tomography. PLoS Comput Biol 2020, 16, e1007962. 32776920 10.1371/journal.pcbi.1007962 PMC7444595

[36] Lucic V, Fernandez-Busnadiego R, Laugks U, Baumeister W: Hierarchical detection and analysis of macromolecular complexes in cryo-electron tomograms using Pyto software. J Struct Biol 2016, 196:503–514. 27742578 10.1016/j.jsb.2016.10.004

[37] Rigort A, Gunther D, Hegerl R, Baum D, Weber B, Prohaska S, Medalia O, Baumeister W, Hege HC: Automated segmentation of electron tomograms for a quantitative description of actin filament networks. J Struct Biol 2012, 177:135–144. 21907807 10.1016/j.jsb.2011.08.012

[R38] Barad BA, Medina M, Fuentes D, Wiseman RL, Grotjahn DA: Quantifying organellar ultrastructure in cryo-electron tomography using a surface morphometrics pipeline. J Cell Biol 2023, 222. 10.1083/jcb.202204093 PMC9960335 36786771

[39] Ishemgulova A, Mukhamedova L, Trebichalska Z, Rajecka V, Payne P, Smerdova L, Moravcova J, Hrebik D, Buchta D, Skubnik K, : Endosome rupture enables enteroviruses from the family Picornaviridae to infect cells. Commun Biol 2024, 7:1465. 39511383 10.1038/s42003-024-07147-9 PMC11543853

[40] Antonova D, Nichiporenko A, Sobinina M, Wang Y, Vishnyakov IE, Moiseenko A, Kurdyumova I, Chesnokov YM, Stepanchikova E, Bourkaltseva M, : Genomic transfer via membrane vesicle: a strategy of giant phage phiKZ for early infection. J Virol 2024, 98, e0020524. 39258909 10.1128/jvi.00205-24 PMC11494934

[R41] Hou Z, Shen Y, Fronik S, Shen J, Shi J, Xu J, Chen L, Hardenbrook N, Thompson C, Neumann S, : Correlative in situ cryo-ET reveals cellular and viral remodeling associated with selective HIV-1 core nuclear import. bioRxiv 2025, 10.1101/2025.03.04.641496:2025.2003.2004.641496.

[42] Kreysing JP, Heidari M, Zila V, Cruz-Leon S, Obarska-Kosinska A, Laketa V, Rohleder L, Welsch S, Kofinger J, Turonova B, : Passage of the HIV capsid cracks the nuclear pore. Cell 2025, 188:930–943 e921. 39826544 10.1016/j.cell.2024.12.008

[43] Dahmane S, Schexnaydre E, Zhang J, Rosendal E, Chotiwan N, Kumari Singh B, Yau WL, Lundmark R, Barad B, Grotjahn DA, : Cryo-electron tomography reveals coupled flavivirus replication, budding and maturation. bioRxiv 2024, 10.1101/2024.10.13.618056. PMC12824359 41559045

[44] Dahmane S, Shankar K, Carlson LA: A 3D view of how enteroviruses hijack autophagy. Autophagy 2023, 19:2156–2158. 36471479 10.1080/15548627.2022.2153572 PMC10283406

[45] Ben Chaabene R, Martinez M, Bonavoglia A, Maco B, Chang YW, Lentini G, Soldati-Favre D: Toxoplasma gondii rhoptry discharge factor 3 is essential for invasion and microtubule-associated vesicle biogenesis. PLoS Biol 2024, 22, e3002745. 39137211 10.1371/journal.pbio.3002745 PMC11343613

[46] Xu P, Schumacher D, Liu C, Harms A, Dickmanns M, Beck F, Plitzko JM, Baumeister W, Sogaard-Andersen L: In situ architecture of a nucleoid-associated biomolecular co-condensate that regulates bacterial cell division. Proc Natl Acad Sci U S A 2025, 122, e2419610121. 39739804 10.1073/pnas.2419610121 PMC11725790

[47.*] Zhang X, Sridharan S, Zagoriy I, Eugster Oegema C, Ching C, Pflaesterer T, Fung HKH, Becher I, Poser I, Muller CW, : Molecular mechanisms of stress-induced reactivation in mumps virus condensates. Cell 2023, 186:1877–1894 e1827. 37116470 10.1016/j.cell.2023.03.015 PMC10156176

[48] Liu Y, Zhai H, Alemayehu H, Boulanger J, Hopkins LJ, Borgeaud AC, Heroven C, Howe JD, Leigh KE, Bryant CE, : Cryo-electron tomography of NLRP3-activated ASC complexes reveals organelle co-localization. Nat Commun 2023, 14: 7246. 37945612 10.1038/s41467-023-43180-8 PMC10636019

[49] Wang J, Wu M, Magupalli VG, Dahlberg PD, Wu H, Jensen GJ: Human NLRP3 inflammasome activation leads to formation of condensate at the microtubule organizing center. bioRxiv 2024, 10.1101/2024.09.12.612739. PMC13025127 41894512

[50] Hickey KL, Panov A, Whelan EM, Schafer T, Mizrak A, Kopito RR, Baumeister W, Fernandez-Busnadiego R, Harper JW: Temporal control of acute protein aggregate turnover by UBE3C and NRF1-dependent proteasomal pathways. Proc Natl Acad Sci U S A 2024, 121, e2417390121. 39636856 10.1073/pnas.2417390121 PMC11648907

[51] Zhao DY, Bauerlein FJB, Saha I, Hartl FU, Baumeister W, Wilfling F: Autophagy preferentially degrades non-fibrillar polyQ aggregates. Mol Cells 2024, 84:1980–1994 e1988. 10.1016/j.molcel.2024.04.018 38759629

[52] Hoyer MJ, Capitanio C, Smith IR, Paoli JC, Bieber A, Jiang Y, Paulo JA, Gonzalez-Lozano MA, Baumeister W, Wilfling F, : Combinatorial selective ER-phagy remodels the ER during neurogenesis. Nat Cell Biol 2024, 26:378–392. 38429475 10.1038/s41556-024-01356-4 PMC10940164

[53] Li M, Tripathi-Giesgen I, Schulman BA, Baumeister W, Wilfling F: In situ snapshots along a mammalian selective autophagy pathway. Proc Natl Acad Sci U S A 2023, 120, e2221712120. 36917659 10.1073/pnas.2221712120 PMC10041112

[54] Held RG, Liang J, Brunger AT: Nanoscale architecture of synaptic vesicles and scaffolding complexes revealed by cryo-electron tomography. Proc Natl Acad Sci U S A 2024, 121, e2403136121. 38923992 10.1073/pnas.2403136121 PMC11228483

[55] Papantoniou C, Laugks U, Betzin J, Capitanio C, Ferrero JJ, Sanchez-Prieto J, Schoch S, Brose N, Baumeister W, Cooper BH, : Munc13- and SNAP25-dependent molecular bridges play a key role in synaptic vesicle priming. Sci Adv 2023, 9, eadf6222. 37343100 10.1126/sciadv.adf6222 PMC10284560

[56.*] Matsui A, Spangler C, Elferich J, Shiozaki M, Jean N, Zhao X, Qin M, Zhong H, Yu Z, Gouaux E: Cryo-electron tomographic investigation of native hippocampal glutamatergic synapses. eLife 2024, 13. 10.7554/eLife.98458 PMC11534335 39495821

[57] Gilbert MAG, Fatima N, Jenkins J, O’Sullivan TJ, Schertel A, Halfon Y, Wilkinson M, Morrema THJ, Geibel M, Read RJ, : CryoET of beta-amyloid and tau within postmortem Alzheimer’s disease brain. Nature 2024, 631:913–919. 38987603 10.1038/s41586-024-07680-x PMC11269202

[58] Rodrigues-Oliveira T, Wollweber F, Ponce-Toledo RI, Xu J, Rittmann SKR, Klingl A, Pilhofer M, Schleper C: Actin cytoskeleton and complex cell architecture in an Asgard archaeon. Nature 2023, 613:332–339. 36544020 10.1038/s41586-022-05550-y PMC9834061

[59.*] Pi H, Sun R, McBride JR, Kruse ARS, Gibson-Corley KN, Krystofiak ES, Nicholson MR, Spraggins JM, Zhou Q, Skaar EP: Clostridioides difficile ferrosome organelles combat nutritional immunity. Nature 2023, 623:1009–1016. 37968387 10.1038/s41586-023-06719-9 PMC10822667

[60] Shimakawa G, Demulder M, Flori S, Kawamoto A, Tsuji Y, Nawaly H, Tanaka A, Tohda R, Ota T, Matsui H, : Diatom pyrenoids are encased in a protein shell that enables efficient CO(2) fixation. Cell 2024, 187:5919–5934 e5919. 39357521 10.1016/j.cell.2024.09.013

[61.*] Xing H, Taniguchi R, Khusainov I, Kreysing JP, Welsch S, Turonova B, Beck M: Translation dynamics in human cells visualized at high resolution reveal cancer drug action. Science 2023, 381:70–75. 37410833 10.1126/science.adh1411

[62] Fedry J, Silva J, Vanevic M, Fronik S, Mechulam Y, Schmitt E, des Georges A, Faller WJ, Forster F: Visualization of translation reorganization upon persistent ribosome collision stress in mammalian cells. Mol Cells 2024, 84:1078–1089 e1074. 10.1016/j.molcel.2024.01.015 PMC7615912 38340715

[63] Xue L, Spahn CMT, Schacherl M, Mahamid J: Structural insights into context-dependent inhibitory mechanisms of chloramphenicol in cells. Nat Struct Mol Biol 2025, 32:257–267. 39668257 10.1038/s41594-024-01441-0 PMC11832420

[R64] Wagner J, Carvajal AI, Bracher A, Beck F, Wan W, Bohn S, Korner R, Baumeister W, Fernandez-Busnadiego R, Hartl FU: Visualizing chaperonin function in situ by cryo-electron tomography. Nature 2024, 633:459–464. 39169181 10.1038/s41586-024-07843-w PMC11390479

[65] Xing H, Rosenkranz RRE, Rodriguez-Aliaga P, Lee TT, Majtner T, Bohm S, Turonova B, Frydman J, Beck M: In situ analysis reveals the TRiC duty cycle and PDCD5 as an open-state cofactor. Nature 2025, 637:983–990. 39663456 10.1038/s41586-024-08321-z PMC11754096

[66] Singh D, Soni N, Hutchings J, Echeverria I, Shaikh F, Duquette M, Suslov S, Li Z, van Eeuwen T, Molloy K, : The molecular architecture of the nuclear basket. Cell 2024, 187:5267–5281 e5213. 39127037 10.1016/j.cell.2024.07.020 PMC11416316

[67.*] Tamborrini D, Wang Z, Wagner T, Tacke S, Stabrin M, Grange M, Kho AL, Rees M, Bennett P, Gautel M, : Structure of the native myosin filament in the relaxed cardiac sarcomere. Nature 2023, 623:863–871. 37914933 10.1038/s41586-023-06690-5 PMC10665186

[68] Tollervey F, Rios MU, Zagoriy E, Woodruff JB, Mahamid J: Molecular architectures of centrosomes in C. elegans embryos visualized by cryo-electron tomography. Dev Cell 2024, 10.1016/j.devcel.2024.12.002. PMC11948214 39721584

[69] Chakraborty S, Martinez-Sanchez A, Beck F, Toro-Nahuelpan M, Hwang IY, Noh KM, Baumeister W, Mahamid J: Cryo-ET suggests tubulin chaperones form a subset of microtubule lumenal particles with a role in maintaining neuronal microtubules. Proc Natl Acad Sci U S A 2025, 122, e2404017121. 39888918 10.1073/pnas.2404017121 PMC11804619

[70] Akõl C, Xu J, Shen J, Zhang P: Unveiling the complete spectrum of SARS-CoV-2 fusion stages by in situ cryo-ET. bioRxiv 2025, 10.1101/2025.02.25.640151:2025.2002.2025.640151. PMC12134289 40461447

[71] Hernandez-Gonzalez M, Calcraft T, Nans A, Rosenthal PB, Way M: A succession of two viral lattices drives vaccinia virus assembly. PLoS Biol 2023, 21, e3002005. 36862727 10.1371/journal.pbio.3002005 PMC10013923

[72] Shah PNM, Gilchrist JB, Forsberg BO, Burt A, Howe A, Mosalaganti S, Wan W, Radecke J, Chaban Y, Sutton G, : Characterization of the rotavirus assembly pathway in situ using cryoelectron tomography. Cell Host Microbe 2023, 31: 604–615 e604. 36996819 10.1016/j.chom.2023.03.004 PMC7615348

[73] Prazak V, Mironova Y, Vasishtan D, Hagen C, Laugks U, Jensen Y, Sanders S, Heumann JM, Bosse JB, Klupp BG, : Molecular plasticity of herpesvirus nuclear egress analysed in situ. Nat Microbiol 2024, 9:1842–1855. 38918469 10.1038/s41564-024-01716-8 PMC7616147

[74.*] Xia X, Sung PY, Martynowycz MW, Gonen T, Roy P, Zhou ZH: RNA genome packaging and capsid assembly of bluetongue virus visualized in host cells. Cell 2024, 187:2236–2249 e2217. 38614100 10.1016/j.cell.2024.03.007 PMC11182334

[75] Ni T, Mendonca L, Zhu Y, Howe A, Radecke J, Shah PM, Sheng Y, Krebs AS, Duyvesteyn HME, Allen E, : ChAdOx1 COVID vaccines express RBD open prefusion SARS-CoV-2 spikes on the cell surface. iScience 2023, 26, 107882. 37766989 10.1016/j.isci.2023.107882 PMC10520439

[76] Watanabe R, Zyla D, Parekh D, Hong C, Jones Y, Schendel SL, Wan W, Castillon G, Saphire EO: Intracellular Ebola virus nucleocapsid assembly revealed by in situ cryo-electron tomography. Cell 2024, 187:5587–5603 e5519. 39293445 10.1016/j.cell.2024.08.044 PMC11455616

[R77] Vallbracht M, Bodmer BS, Fischer K, Makroczyova J, Winter SL, Wendt L, Wachsmuth-Melm M, Hoenen T, Chlanda P: Nucleocapsid assembly drives Ebola viral factory maturation and dispersion. Cell 2025, 188:704–720 e717. 39742805 10.1016/j.cell.2024.11.024

[R78] Klumpe S, Senti KA, Beck F, Sachweh J, Hampoelz B, Ronchi P, Oorschot V, Brandstetter M, Yeroslaviz A, Briggs JAG, : In-cell structure and snapshots of copia retrotransposons in intact tissue by cryoelectron tomography. Cell 2025, 10.1016/j.cell.2025.02.003. 40049165

[79] Lien YW, Amendola D, Lee KS, Bartlau N, Xu J, Furusawa G, Polz MF, Stocker R, Weiss GL, Pilhofer M: Mechanism of bacterial predation via ixotrophy. Science 2024, 386, eadp0614. 39418385 10.1126/science.adp0614

[80] Sharma H, Jespersen N, Ehrenbolger K, Carlson LA, Barandun J: Ultrastructural insights into the microsporidian infection apparatus reveal the kinetics and morphological transitions of polar tube and cargo during host cell invasion. PLoS Biol 2024, 22, e3002533. 38422169 10.1371/journal.pbio.3002533 PMC10931468

[81] Zhu S, Bradfield CJ, Maminska A, Park ES, Kim BH, Kumar P, Huang S, Kim M, Zhang Y, Bewersdorf J, : Native architecture of a human GBP1 defense complex for cell-autonomous immunity to infection. Science 2024, 383, eabm9903. 38422126 10.1126/science.abm9903 PMC12091997

[82] Sun Y, Sheng Y, Ni T, Ge X, Sarsby J, Brownridge PJ, Li K, Hardenbrook N, Dykes GF, Rockliffe N, : Rubisco packaging and stoichiometric composition of the native β-carboxysome in Synechococcus elongatus PCC7942. Plant Physiol 2024, 197. 10.1093/plphys/kiae665 PMC11973430 39680612

[83] Kong WW, Zhu Y, Zhao HR, Du K, Zhou RQ, Li B, Yang F, Hou P, Huang XH, Chen Y, : Cryo-electron tomography reveals the packaging pattern of RuBisCOs in Synechococcus beta-carboxysome. Structure 2024, 32:1110–1120 e1114. 38823379 10.1016/j.str.2024.05.007

[84] Ni T, Sun Y, Burn W, Al-Hazeem MMJ, Zhu Y, Yu X, Liu LN, Zhang P: Structure and assembly of cargo Rubisco in two native alpha-carboxysomes. Nat Commun 2022, 13:4299. 35879301 10.1038/s41467-022-32004-w PMC9314367

[85] Elad N, Hou Z, Dumoux M, Ramezani A, Perilla JR, Zhang P: In-cell structure and variability of pyrenoid Rubisco. bioRxiv 2025, 10.1101/2025.02.27.640608:2025.2002.2027.640608. PMC12368222 40835820

[86] Wang C, Jiang W, Leitz J, Yang K, Esquivies L, Wang X, Shen X, Held RG, Adams DJ, Basta T, : Structure and topography of the synaptic V-ATPase-synaptophysin complex. Nature 2024, 631:899–904. 38838737 10.1038/s41586-024-07610-x PMC11269182

[R87] Waltz F, Righetto RD, Lamm L, Salinas-Giege T, Kelley R, Zhang X, Obr M, Khavnekar S, Kotecha A, Engel BD: In-cell architecture of the mitochondrial respiratory chain. Science 2025, 387:1296–1301. 40112058 10.1126/science.ads8738

[R88] Muhleip A, Flygaard RK, Baradaran R, Haapanen O, Gruhl T, Tobiasson V, Marechal A, Sharma V, Amunts A: Structural basis of mitochondrial membrane bending by the I-II-III(2)-IV(2) supercomplex. Nature 2023, 615:934–938. 36949187 10.1038/s41586-023-05817-y PMC10060162

[89] Cassidy CK, Qin Z, Frosio T, Gosink K, Yang Z, Sansom MSP, Stansfeld PJ, Parkinson JS, Zhang P: Structure of the native chemotaxis core signaling unit from phage E-protein lysed E. coli cells. mBio 2023, 14, e0079323. 37772839 10.1128/mbio.00793-23 PMC10653900

[90] Zhu Y, Koo CW, Cassidy CK, Spink MC, Ni T, Zanetti-Domingues LC, Bateman B, Martin-Fernandez ML, Shen J, Sheng Y, : Structure and activity of particulate methane monooxygenase arrays in methanotrophs. Nat Commun 2022, 13:5221. 36064719 10.1038/s41467-022-32752-9 PMC9445010

[91] Lange F, Ratz M, Dohrke JN, Le Vasseur M, Wenzel D, Ilgen P, Riedel D, Jakobs S: In situ architecture of the human prohibitin complex. Nat Cell Biol 2025, 27:633–640. 40119201 10.1038/s41556-025-01620-1 PMC11991916

[R92] Hou Z, Nightingale F, Zhu Y, MacGregor-Chatwin C, Zhang P: Structure of native chromatin fibres revealed by Cryo-ET in situ. Nat Commun 2023, 14:6324. 37816746 10.1038/s41467-023-42072-1 PMC10564948

[93] McCafferty CL, Klumpe S, Amaro RE, Kukulski W, Collinson L, Engel BD: Integrating cellular electron microscopy with multimodal data to explore biology across space and time. Cell 2024, 187:563–584. 38306982 10.1016/j.cell.2024.01.005

[94] Costa-Filho JI, Theveny L, de Sautu M, Kirchhausen T: Cryo-Samba: self-supervised deep volumetric denoising for cryo-electron tomography data. J Struct Biol 2024, 217, 108163. 39710216 10.1016/j.jsb.2024.108163 PMC11908917

[95.*] Heebner JE, Purnell C, Hylton RK, Marsh M, Grillo MA, Swulius MT: Deep learning-based segmentation of cryo-electron tomograms. J Vis Exp 2022, 10.3791/64435. 36440884

[R96] Eisenstein F, Fukuda Y, Danev R: Smart parallel automated cryo-electron tomography. Nat Methods 2024, 21:1612–1615. 39117874 10.1038/s41592-024-02373-9

[97.*] Ermel U, Cheng A, Ni JX, Gadling J, Venkatakrishnan M, Evans K, Asuncion J, Sweet A, Pourroy J, Wang ZS, : A data portal for providing standardized annotations for cryo-electron tomography. Nat Methods 2024, 21:2200–2202. 39433879 10.1038/s41592-024-02477-2

